# Loneliness in mid-life and older adults from ethnic minority communities in England and Wales: measure validation and prevalence estimates

**DOI:** 10.1007/s10433-020-00564-9

**Published:** 2020-04-07

**Authors:** Christina R. Victor, Christine Dobbs, Kenneth Gilhooly, Vanessa Burholt

**Affiliations:** 1 Department of Clinical Sciences, Brunel University London, Kingston Lane, Uxbridge, UB8 7PH UK; 2 Centre for Innovative Ageing, Swansea University, Singleton Park, Swansea, SA2 8PP UK; 3 Faculty of Medical and Health Sciences, The University of Auckland, Auckland, New Zealand

**Keywords:** Loneliness, Ethnic minority groups, Older adults

## Abstract

We investigated the prevalence of loneliness among 1206 adults aged 40 + from six minority communities in England and Wales: Black Caribbean, Black African, Indian, Pakistani, Bangladeshi and Chinese. Replicating the approach from the previous studies, we demonstrate robust acceptability, reliability and validity for both the six-item De Jong Gierveld (DJG) and single-item loneliness scales in our six ethnic groups. The prevalence of loneliness using a single-item question (loneliness reported as often/always) ranges from 5% (Indian) to 14% (Chinese) compared with approximately 5% for the general population aged 40 + in Britain. Levels of loneliness are very much higher using the DJG scale. Using a loneliness threshold score of 5 +, the percentage ranged from 13% (Indian) to 36% (Chinese). We explored the importance of six established loneliness vulnerability factors for our sample using regression modelling. Three factors were not associated with loneliness—number of children, gender and health rating, and three factors were protective: younger age, being married and low financial strain. The addition of ethnicity did not change these relationships or enhance statistical power of our models. Being a member of the African Caribbean group was protective against loneliness but not for the other groups included in our study. We suggest that exposure to loneliness vulnerability factors rather than ethnicity per se or measurement artefact underpins differences in loneliness across ethnic groups.

**Electronic supplementary material:**

The online version of this article (10.1007/s10433-020-00564-9) contains supplementary material, which is available to authorized users.

## Introduction

The population of mid and later life adults in Europe is becoming increasingly diverse in terms of ethnicity. This reflects the ageing of the cohorts of post-second world war transnational migrants and their families who came to Europe during the three decades 1950–1980. Whilst the nature of the specific ethnic or migrant groups may vary across different European countries, this axis of heterogeneity is becoming increasing important in terms of gerontological research, policy and practice. Torres (2015, 2019) and Phillipson (2015) argue that ethnogerontology is an important but embryonic field of study. Gerontology needs to embrace the diversity characterising the mid and later life populations of our continent if we are to undertake research which fully captures the experience of ageing in the twenty-first century. We suggest there are five areas of activity that needed to be included in a research agenda which will fully capture the complexity of the experience of mid and later life adult for ethnic minority groups. First, we need to systematically look at within-group variations in terms of cohort migration experience (Victor 2015) and adopt an inter-sectional approach which embraces age, gender and social position. Second, we need to compare experiences across different ethnic groups to establish the similarities/differences between them in terms of experiences of mid and later life (Burholt et al. 2016; Victor et al. 2019; Evandrou et al. 2016; El Fakiri et al. 2017). Thirdly, we need to adopt an (inter)national perspective to examine the experiences of specific ethnic minority groups in different countries such as Indians moving to Britain, Canada or Australia (Victor 2015) and draw comparisons between ethnic groups and their peers in their country of origin (Victor 2015). Fourthly, we need to move away from a problem-centred approach and develop a more expansive research agenda which encompasses well-being and broader experiences of ageing and later life within a life course context. Finally, to undertake these activities, we need to develop a suite of methods and measures that are appropriate for use with diverse populations so that we can be assured of the robustness of observed differences across and within groups. This paper addresses two elements of this research agenda. Using the example of loneliness, we explore the utility of two standard measurement tools and the prevalence of loneliness for mid-life and older adults from six different ethnic minority groups resident in England and Wales.

## Loneliness and ethnic minority groups

Loneliness is the negative outcome of the discrepancy between an individual’s expectations and actuality of their quality and/or quantity, and potentially mode (in person, online) of social relationships. Loneliness is a key driver of well-being in mid and later life and has been linked with a wide range of negative health outcomes (see, for example, Valtorta et al. 2016). However, in the plethora of literature investigating loneliness in later life, there are few studies that report prevalence for mid-life and older adults from ethnic minority groups either in terms of studies of single minority groups, comparisons across ethnic groups and comparisons with native-born peers in the host country or peers in the country of origin. A further complexity in evaluating and interpreting research in this area is that studies may report loneliness outcomes for ethnic minority groups, linguistic groups or by migrant status where presumptions are made about ethnic/linguistic characteristics of participants.

There are three potential factors that may suggest that loneliness would be higher in minority and/or migrant communities: (a) cultural; (b) differential exposure to loneliness vulnerability factors and (c) measurement artefact. Loneliness is the expression of the deficit in desired and experienced quantity/quality of social relationships. These expectations are culturally located (Jylhä and Jokela 1990) and may be compromised by living in a foreign culture (Lykes and Kemmelmeier 2014; Van Staden and Coetzee 2010). Older and mid-life adults who have migrated to another country may, therefore, be more vulnerable to experiencing loneliness than either their native-born peers or those in their country of origin because of compromised social norms. This explanation has largely been explored quantitatively by studies comparing loneliness across different migrant groups and native-born peers as exemplified by two Canadian studies comparing loneliness among older migrants and native-born Canadians. Wu and Penning (2015) compared levels of loneliness for immigrants to Canada aged 60 + from Europe, China and South Asia with the Canadian-born population using the 2007 Canadian General Social survey. Loneliness was measured using the eleven-item De Jong Gierveld scale (De Jong and Kamphuis 1985). First-generation migrants, especially those of non-European, Chinese or South Asian origins, reported significantly higher levels of loneliness than native-born Canadians after adjustment for health and income factors. De Jong et al. (2015) differentiated between native-born Canadians aged 65 + and those born in Europe (France or Britain), other European Countries and those born elsewhere. Using the six-item De Jong Gierveld (DJG) scale, mean loneliness scores were significantly different across these four groups ranging from 1.26 for native Canadians, 1.29 for those born in Britain or France, 1.54 for those born elsewhere in Europe and 1.93 for those born elsewhere. Both studies suggest that migrants who share a common language with the receiving country have similar levels of loneliness to their native-born peers, whilst those where these two factors are not congruent have higher levels of loneliness. A key caveat to these conclusions is the reliance on implicit assumptions that linguistic congruence with the host country is acting as a proxy for cultural congruence which is fully acknowledged and which requires further investigation.

It is plausible that variations in loneliness across different ethnic/migrant groups may reflect differential exposure to loneliness vulnerability factors such as widowhood, low income or poor health. Fokkema and Naderi (2013) explored this explanation in their study which compared levels of loneliness for older Turks (50–79) living in Germany with native Germans peers. They reported a significantly higher crude prevalence of loneliness for the Turkish participants as measured by a score of 2 + on the De Jong Gierveld six-item scales (54% vs. 43%) and a higher mean loneliness score (2.1 vs. 1.6-range 0–6). They concluded the higher crude rates of loneliness among the Turkish were explained by their poorer health and lower socio-economic status rather than language or cultural issues ethnicity. That rates of loneliness varying across and within ethnic minority groups evidenced by a comparative study of older adults from six different ethnic groups in Britain reported high levels of loneliness using a five-point response to a single-item loneliness question (never, rarely, sometimes, often and most of the time). Between 24 and 48% of those aged 65 + from Pakistani, Bangladeshi, African Caribbean and Chinese groups report being lonely/often or always. In contrast, the Indian group reported levels of loneliness that matched that of the general population with approximately 10% reporting that they were often or always lonely (Victor et al. 2012). The prevalence of loneliness among the Indian group mirrors that for the general population (Victor et al. 2012; Victor 2015).

Observed high levels of loneliness among minority elders may reflect issues relating to the appropriateness of our measurement tools in terms of translation, acceptability, reliability and validity. The two main approaches to the translation of loneliness questions are forward translation (Fokkema and Naderi 2013) or forward/backward translation as used by Victor (2015). There are a number of studies that have focused upon translating the DJG scale for individual languages (e.g. Çavdar et al. 2015). De Jong Gierveld and Van Tilburg (2010) evaluate the use of the six-item DJG scale in 7 different countries France, Germany, The Netherlands, Russia, Bulgaria, Georgia and Japan and consider the translated scales to be both reliable and valid. Uysal-Bozkir et al. (2017) validated a translated version of the eleven-item DJG scale for use with Turkish and Moroccan elders and a Dutch version for Surinamese elders. The emphasis in this paper is upon measure validation, but the authors report high levels of loneliness for Turkish participants (24%) compared with Moroccan (12%), Surinamese (13%) or Dutch (6%). Van Tilburg and Fokkema (2020) used the Longitudinal Ageing Study Amsterdam to consider two different explanations for the higher levels of loneliness observed among older Turkish and Moroccan migrants living in The Netherlands compared to Dutch-born peers. The two explanations investigated were: (a) differential exposure to loneliness vulnerability factors or (b) measurement artefact resultant from variations in how loneliness was understood and experienced. They concluded that the observe differences were not a measurement artefact and that these were attenuated, but not eradicated, when a range of loneliness vulnerability measures (social participation, depressive symptoms, satisfaction with material resources and psychological factors) were considered.

Our paper has two aims. First we focus upon establishing the veracity of two measures of loneliness—the six-item DJG scale and a single-item loneliness question for mid-life and older adults (aged 40 +) from the six largest minority groups in England and Wales. We first consider the acceptability, reliability and validity of our approach to measuring loneliness across the six minority groups. We replicate the approaches described by De Jong Gierveld and Van Tilburg (2010) and Uysal-Bozkir et al. (2017) which consisted of (a) forward/back translation of loneliness questions; (b) evaluation of acceptability of questions; (c) reliability and (d) validity. This enables us to compare the technical properties of measure performance for our populations with the existing literature. Second, we undertake an exploratory analysis of loneliness prevalence and examine the importance of loneliness vulnerability factors and ethnicity in explaining observed loneliness prevalence.

## Methods

### Source of data

Our data are derived from a study on inter- and intra-generational and transnational caring among the largest minority groups within England and Wales: Indian, Pakistani, Bangladeshi, African Caribbean, Black African and Chinese populations (see Ahmet and Victor 2015; Burholt et al. 2016, 2018; Victor et al. 2019). The primary focus of this study was on these six minority groups and did not include participants from other groups such as migrants from Eastern Europe or Ireland. Consequently, the findings reported in this paper are not a full comparison of all ethnic groups in England and Wales.

Full details of the sampling procedures and detailed non-response data are published (Victor et al. 2019). In summary, this study used face-to-face interviews with 1206 adults aged 40 + conducted in Punjabi and Urdu (Pakistani group), Gujarati and Hindi (Indian group), Mandarin Chinese, Bengali (Bangladeshi), Somali, Yoruba or Urdu (Black African) and English by a specialist research group (*Ethnic Focus*) between October 2011 and April 2012. This survey examined the prevalence of informal care among six major minority ethnic groups (Victor et al. 2019). The target sample size was 1200 (200 persons per ethnic group divided into 100 aged 40–64 and 100 aged 65 +). Our sample was drawn using information from the 30,000 Postcode Address file (PAF) units into which England and Wales is subdivided. The use of these geographical units to draw population samples is standard practice for many large-scale surveys in the UK. Each PAF was ranked separately for Wales and England for each ethnic group, creating 12 lists from which systematic random samples were drawn to select sampling points. Potential participants approached by Ethnic Focus who did not self-identify with one of the six ethnic groups were ineligible for inclusion in the study. The overall response rate for the study was 38% with variations across groups. Indian participants had the highest response rate (48%), and Chinese the lowest (23%). The other response rates were as follows: Black Caribbean, 40%; Black African, 38%; Pakistani, 43%; and Bangladeshi, 39%.

### Methods and measures

#### Study population

All those who participated in the index study are included in our analysis as we wanted to include both older adults, those currently experiencing later life, and those in mid-life who will be the elders of the future. This both enhances the power of study in both statistical and development of tools and measures appropriate for the future ageing population.

#### Loneliness

Our survey included both a single-item loneliness question and the six-item De Jong Gierveld (DJG) scale. The question asked ‘how often do you feel lonely’ with a five-point response scale (never, rarely, sometimes, often and most of the time) and is broadly comparable with the UK Government (Office of National Statistics 2018) recommended loneliness measure. We followed their protocol and created a dichotomous variable that differentiated those reporting loneliness (responses of often/most of the time) and those not reporting loneliness (responses never, rarely and sometimes). This replicates the approach of Victor et al. (2012) and enables us to consider the prevalence of loneliness across our different ethnic groups and with the existing literature and nation estimates for the UK. The DJG scale, as well providing a total score (range 0–6), differentiates between emotional and social loneliness both with a range of 0–3. Mean scores are computed, and the total score may also be divided into sub-categories as specified by the scale developers: 0–1 not lonely; 2–4 intermediate loneliness and 5 + severe loneliness or dichotomised between lonely (a score of 2 +) or not lonely (< 2) (Van Tilburg 1999). We use both mean scores and the categorical groupings in our paper as presentation of prevalence rates of loneliness/severe loneliness provides a public health perspective on the extent of loneliness across and within groups.

#### Measure evaluation

To the best of our knowledge, there are few examples of the formal evaluation of self-rating loneliness scales across different linguistic and ethnic groups. The congruence between the single-item loneliness question and the eleven-item DJG scale has been evaluated in a sample of British and Australian elders (Victor et al. 2005). The DJG scale has been translated into Turkish and Moroccan (Uysal-Bozkir et al. (2017) as well as Chinese, Russian, French, German, Georgian, Bulgarian and Japanese (De Jong Gierveld and Van Tilburg 2010). When we undertook our study, the DJG measure was only available for Chinese (Leung et al. 2008) but not available for the languages used by our participants: Punjabi and Urdu (Pakistani group), Gujarati and Hindi (Indian group), Mandarin Chinese, Bengali (Bangladeshi), Somali, Yoruba (Black African) and Urdu (Pakistani group). We undertook an evaluation of the acceptability, reliability and validity of these two measures in our six populations.

#### Acceptability of loneliness measures

We used established best practice, front–back translation methods using bilingual/multi-lingual research and fieldwork staff and university students, to translate the DJG and single-item loneliness question (seven questions in total) into the eight languages used by our participant groups. The questions were piloted prior to the main fieldwork. No specific modifications or adaptations were made to the questions, and no difficulties were reported with the ‘abstract concepts’ such as a general sense of emptiness, the first-person nature of the questions, or with responding to the self-rated loneliness question. Acceptability in the main study was evaluated using two indicators: (a) participants declining to answer some or all the questions; (b) reports from fieldworkers of interviews being terminated when these questions were asked.

#### Reliability and validity of loneliness measures

We examine the reliability and validity of our loneliness measures using the approaches described by De Jong Gierveld and Van Tilburg (2010) for the six-item version and Uysal-Bozkir et al. (2017) for the eleven-item version. This enables us to directly compare the performance of these in our populations with the existing literature. Neither study tested differential item functioning to examine potential differences in the way the different items in the DJG scale functioned. We tested this using six different regression analyses for each DJG scale item as the dependent variable and total DJG score and ethnicity as independent variables.

Internal reliability for the DJG scale is evaluated by Cronbach’s alpha (α) for each ethnic group. Following the approach of Uysal-Bozkir et al. (2017) and to establish comparability of approach, a score of 0.70 or greater was considered satisfactory for purposes of group comparisons. We also established the correlation between the sub-scales for each group. In the absence of a gold standard, establishing validity is challenging. We replicate the approach of Uysal-Bozkir et al. (2017) and evaluate construct validity for the two-dimensional structure of the DJG scale for each group using confirmatory factor analysis, testing the correlation of the scales with one established loneliness risk factor, life satisfaction and two unrelated measures (number of languages proficient in and the role of the state in paying for care).

We adopted the approach of De Jong Gierveld and Van Tilburg (2010) to test the robustness of established loneliness predictors across our six groups. These authors use six key factors in their analysis testing the performance of the DJG scale across seven different countries: demographic (age, gender), availability of social support (partner status as measured by living with a spouse in the same household and number of living children differentiating those with up to two children and those with three or more children), health (how is your health in general on a five-point scale ranging from ‘very poor’ to ‘very good’) and financial strain (how well participants felt they were making ends meet on a six-point scale from ‘with great difficulty’ to ‘very easily’). We replicated three of these variables: age, gender and number of children. For partner status, self-rated health and financial security, we adapted our measures to establish a best match with those used by De Jong Gierveld and Van Tilburg (2010). We used marital status, dichotomised between those who were married from those who were single, divorced or widowed to represent partner status. In our data set, all of those who were married were living with their spouse, thereby replicating the measure of De Jong Gierveld and Van Tilburg (2010). Our measure of overall health used a three-point scale ranging from good to not good rather than a five-point scale and is dichotomised into good health versus not good/fair health. Financial security was measured by how well participants needs were met by their financial resources. We distinguished those whose needs were poorly met from those who needs were fairly or well met on a three-point scale (ranging from cannot make payments, can barely meet payments to easily make payments). We operationalised financial strain as those who could not or barely meet payments. We use binary logistic regression to test the veracity of these six key predictors of loneliness across our key ethnic groups. We present two models one with and without ethnicity as a predictor variable which enables us to examine the importance of ethnicity as an independent loneliness predictor. Statistical significance is attributed to those differences robust at *p* = 0.01.

## Results

We present our results in three sections: (a) the characteristics of our analytical sample; (b) acceptability, reliability and validity of our two loneliness measures across each group and (c) the prevalence of loneliness by ethnic group and the relationship between loneliness, key predictor variables and ethnicity.

### Characteristics of the analytic sample

We recruited 1206 participants from six ethnic groups (Black Caribbean, Black African, Chinese, Indian, Bangladeshi and Pakistani). The mean age of our sample was 65 with no statistically significant differences between groups. Our Indian participants were significantly more likely to report that their financial resources meet their needs compared with other groups and had significantly better health. The variations in the proportion of those who are divorced/widowed as well as differences in gender proportions between groups, were not statistically significant (Table 1).

**Table 1:** Characteristics of the sample by ethnic group

	Black Caribbean	Black African	Indian	Pakistani	Bangladeshi	Chinese	Significant Chi Square
*N*	224	215	201	211	199	156	
Mean age (maximum)	66 (85)	65 (78)	66 (92)	66 (82)	65 (78)	66 (89)	
% Male	47	49	50	54	50	57	
% Married	50	55	68	72	71	67	37.1 *p *= 0.001
% With 3 + children	50	59	41	56	63	24	70.01 *p *= 0.001
Mean life satisfaction score (range 5–35)	21	21	26	23	20	21	
% Financial strain	30	42	5	28	41	29	87.8 *p *= 0.00
% Without long standing limiting illness	72	70	71	66	67	74	
% Health rated as not good	50	57	51	60	73	46	38.3 *p *= 0.00
% With 2 + languages	3	48	55	55	47	54	42.1 *p *= 0.00
% Agree family should pay for care of older relatives	51	47	71	46	32	42	67.2 *p *= 0.00

### Acceptability, reliability and validity of loneliness measures

There were no missing data for either the single-item question or the six-item DJG enabling us to calculate the full scale and two sub-scales for each participant. There were no reports from the field staff of any interviews being terminated because of these questions being asked or negative feedback from participants. Testing for differential item functioning did not reveal any significant variations across the six groups.

We tested the reliability of the DJG scale for each ethnic group using internal consistency as measured by Cronbach’s alpha. The a priori threshold to determine satisfactory performance, a coefficient of 0.7 (or greater), was exceeded for all groups for both full scale and sub-scales with one exception: emotional loneliness for the Black African ethnic group where it was close to this value (0.68) (Table 2). For three of our groups, Black Caribbean, Indian and Chinese, the reliability ratings were above 0.8 for all three elements of the DJG scale.

**Table 2:** Reliability tests (Cronbach’s alpha) on De Jong Gierveld scale: total, emotional and social loneliness scales (*cf* Uysal-Bozkir et al. 2017, Table 2)

Scale	Total	Black C.	Black A.	Indian	Pakistani	Bangladeshi	Chinese
*N*	1206	224	215	201	211	199	156
Total loneliness score	0.84	0.87	0.77	0.81	0.86	0.82	0.86
Emotional loneliness sub-scale	0.79	0.84	0.68	0.81	0.79	0.76	0.82
Social loneliness sub-scale	0.86	0.84	0.84	0.89	0.89	0.81	0.89

We evaluated the relationship between the single-item loneliness question with the total DJG score and two sub-scales. Correlations between the measures are robust across all groups with the self-rating question showing stronger correlation with emotional as compared with social loneliness (Table 3).

**Table 3:** Correlation between self-rated loneliness scores and De Jong Gierveld scale scores

DJG Scale	Total	Black C.	Black A.	Indian	Pakistani	Bangladeshi	Chinese
*N*	1206	224	215	201	211	199	156
Total loneliness score	0.60	0.68	0.44	0.58	0.60	0.65	0.61
Emotional loneliness sub-scale	0.66	0.73	0.59	0.74	0.68	0.68	0.65
Social loneliness sub-scale	0.38	0.48	0.44	0.25	0.35	0.45	0.46

All correlations, p < .001

We evaluated the construct validity of the DJG scale by undertaking a confirmatory factor analysis which supported the existence of a two-factor solution across our groups (Table 4). We used three standard measures of fit: (a) comparative fit index (CFI); (b) root mean squared error of approximation (RMSEA) and (c) root mean squared residual (SRMR). All measures of fit met our pre-defined criteria for the total sample and the Black African, Indian and Pakistani groups. The remaining 3 groups (Black Caribbean, Bangladeshi and Chinese) meet two out of three criteria. We conclude that the two-factor solution was acceptable both for the total sample and our six constituent groups (Table 5).

**Table 4:** CFA model fit results for two-factor model of DJG data (*cf*. Uysal-Bozkir et al. 2017, Table 4)

	Total	Black C.	Black A.	Indian	Pakistani	Bangladeshi	Chinese
*N*	1206	224	215	201	211	199	156
CFI (criterion > 0.95)	0.99	.99	1.00	0.99	0.99	0.97	0.98
RMSEA (criterion < .06)	.056	0.07	0.00	0.03	0.02	0.09	0.10
SRMR (criterion < .08)	0.02	0.03	0.02	0.03	0.03	0.05	0.03
Criteria met/3	3/3	2/3	3/3	3/3	3/3	2/3	2/3

*CFI* comparative fit index; *RMSEA* root mean squared error of approximation; *SRMR* root mean squared residual

**Table 5:** Estimated loadings of single DJG scale items on the 2 loneliness sub-scales. (*cf.* Uysal-Bozkir, et al. 2017, Table 5)

	Total	Black C.	Black A.	Indian	Pakistani	Bangladeshi	Chinese
*N*	1206	224	215	201	211	199	156
*Emotional*							
Empty	0.78	0.79	0.69	0.83	0.79	0.93	0.76
Miss	0.71	0.76	0.55	0.80	0.68	0.63	0.81
Rejected	0.74	0.83	0.71	0.66	0.81	0.67	0.75
*Social*							
Lean	0.80	0.70	0.76	0.85	0.88	0.84	0.77
Trust	0.86	0.84	0.86	0.88	0.87	0.79	0.94
Close	0.81	0.87	0.78	0.84	0.82	0.68	0.85

Significant correlations were observed for the DJG scales with the theorised congruent measure (well-being) but not the unrelated variables (fluency in languages and support for the older people in need), except for the Black African group (Table 6).

**Table 6:** Correlations of congruent and non-congruent variables with the total, emotional and social Loneliness scales of DJG scales. (*cf*. Uysal-Bozkir et al. 2017, Table 6)

	All	Black Caribbean.	Black African.	Indian	Pakistani	Bangladeshi	Chinese
*N*	1206	224	215	201	211	199	156
SWLS (5–35)							
Total	− 0.49**	− 0.48**	− 0.38**	− 0.65**	− 0.53**	− 0.44**	− 0.44**
Social	− 0.41**	− 0.46**	− 0.38**	− 0.45**	− 0.49**	− 0.33**	− 0.39**
Emotion	− 0.44**	− 0.40**	− 0.32**	− 0.62**	− 0.43**	− 0.44**	− 0.40**
Who pays (State = 0, Family = 1)							
Total	− 0.06*	0.04	− 0.14*	− 0.09	0.06	0.03	− 0.02
Social	− 0.04	0.09	− 0.09	− 0.15*	− 0.07	0.05	− 0.08
Emotion	− 0.08*	0.01	− 0.15*	− 0.09	− 0.03	0.00	− 0.05
Number of proficient languages							
Total	− 0.08*	− 0.06	− 0.11	− 0.01	− 0.06	− 0.13	0.02
Social	− 0.05	0.01	− 0.02	0.09	− 0.07	− 0.06	0.00
Emotion	− 0.08*	0.09	− 0.16*	0.09	− 0.05	− 0.17	− 0.03

** *p* < .01; * *p* < .05

### The prevalence and predictors of loneliness

Irrespective of the measure used, loneliness was consistently higher in the Black Caribbean, Black African, Pakistani, Bangladeshi and Chinese groups as compared with the Indian group (Table 7). Mean scores on the DJG scale ranged from 1.8 (Indian) to 2.8 (Chinese), and scores for social loneliness were higher than those for emotional loneliness across all groups. For the DJG scale, we used established cut points to distinguish significant loneliness (a score of five or more) or to differentiate the lonely (a score of two or more) from the non-lonely (a score of under two). The prevalence of loneliness was high in our study. Using the DJG scale threshold of a score of two or more, 60% of all participants were defined as lonely with 24% classed as severely lonely: a score of five or more. The single-item loneliness question generated lower prevalence rates with 10% defined as always/often lonely and 38% as always/sometimes/often lonely. There are significant variations in loneliness prevalence across our six groups with our Indian participants reporting the lowest levels of loneliness and our Chinese group the highest. The prevalence of loneliness defined as those who defined themselves as often/always lonely on the single-item question ranged from 5% for our Indian group to 14% for Chinese participants (Chi-square (*df* = 5) = 14.27; *p* = <0.01). The DJG score of five or more for these two groups was 13% and 36%, respectively. Participants from the Indian group reported the lowest levels of loneliness across all measures with the Chinese group reporting the highest level of loneliness across 6 of our 7 measures (Table 7). Detailed age and ethnic group loneliness scores are provided in supplementary Tables S1–S3.

**Table 7:** De Jong Gierveld (DJG) loneliness scale and self-rated loneliness by ethnic group

Scale	Total	Black Caribbean	Black African	Indian	Pakistani	Bangladeshi	Chinese	Chi Square, *df* = 5
	1206	224	215	201	211	199	156	
Mean DJG Total Loneliness score (range 0–6)	2.5	2.6	2.7	1.8	2.4	2.6	2.8	
Mean DJG Emotional loneliness scale score (range 0–3)	1.0	1.2	1.1	0.7	1.0	1.1	1.3	
Mean DJG Social loneliness scale score (range 0–3)	1.4	1.4	1.6	1.1	1.3	1.5	1.4	
% with total DJG Loneliness score of 2+	60	57	70	51	59	63	61	16.36 *p* < .01
% with total DJG Loneliness score of 5+	24	28	21	13	24	26	36	28.96 *p* < .01
% often/always lonely	10	12	7	5	11	11	14	14.27 *p *= <0.1

We undertook two logistic regression models to investigate the relationship between loneliness, using the dichotomised single-item question, and ethnicity (see supplementary Tables 2a and 2b for the full models). In our model, odds ratios less than one indicate a greater risk of loneliness and of more than one as not being lonely. Our first model replicated the approach of De Jong Gierveld and Van Tilburg (2010) to test the robustness of established loneliness predictors across the whole sample, not taking ethnicity into account. Two factors were protective against loneliness: being married and not being financially strained with increased age associated with loneliness vulnerability (Table 8). Our second model added ethnicity as a predictor using the Chinese as the reference group. (They have the highest rate of self-identified loneliness at 14%.) In our second model, we included ethnicity which did not significantly increase the predictive power. For all our ethnic groups compared to the reference category, there is a reduced risk of loneliness, but this is only significant for the Black Caribbean group and the confidence intervals are wide. Full details of the mode are provided in Tables S4 and S5.

**Table 8:** Modelling loneliness and ethnicity: factors protective against loneliness

	Model 1	Odds	95% CI	Model 2	Odds	95% CI
Gender		0.84	0.56–1.27		0.88	0.58–1.33
Age		0.97	0.95–0.99		0.97	0.95–0.99
Married		2.80	1.84–4.25		3.02	1.96–4.65
Number of children		1.48	0.97–2.24		1.37	0.89–2.13
Health rating		1.80	1.08–2.95		1.88	1.13, 3.14
Financial strain		3.33	2.21–5.05		3.28	2.12, 5.07
				Black Caribbean	2.80	1.30–6.02
				Black African	1.58	0.81–3.11
				Indian	2.19	0.94–5.10
				Pakistani	1.19	0.59–2.39
				Bangladeshi	1.64	0.78–3.42
Nagelkerke *R*^2^	.19			.20		

Reference group = Chinese

Improvement in fit, *with* Ethnicity *v*. *without* Ethnicity, *χ*^2^ (5) = 9.94, ns

Coding Key: loneliness group (1 = not lonely; 0 = lonely); total children (1 = 3 or more children; 0 = 2 or fewer); gender (1 = male; 2 = female); age (in years); married (1 = yes; 0 = no); health rating (1 = good; 0 = not good); financial strain (1 = not strained; 0 = strained)

## Discussion

The premise of our paper has been the need for gerontologists develop a research agenda that includes minority elders, recognises the need for comparative studies across and within different ethnic groups and develops appropriate research methods and measures to facilitate these types of studies. We have looked at the acceptability, reliability and validity of two established ways of measuring loneliness: the six-item De Jong Gierveld scale and a single-item loneliness question using a sample of 1206 adults aged 40 + recruited for a study of family caregiving in minority communities in England and Wales (Victor et al. 2019).

It is important to acknowledge the methodological and substantive limitations of our study in term. Methodologically, in terms of measurement evaluation, our data are cross-sectional which precludes establishing the stability and reliability of our loneliness measures using a test–retest approach. Substantively, there were challenges in recruitment to the study. Our sample is large for studies focused upon mid-life and older adults from minority groups in the UK and was generated by a robust fieldwork process. However, our overall response rate was 38%. This is comparable with the 40% reported by Sheldon et al. (2007) in a review of health surveys, but we recognise that there are potential issues around the generalisability of our prevalence estimates because of the response. Establishing the representativeness of our samples in a UK context is challenging because of the established under-representation of ethnic groups in the decennial Census. With this caveat, our samples broadly approximate to the key parameters age, gender, marital status, health and quality of life that characterise the minority communities in the UK. It is important to acknowledge that recruiting older and mid-life adults to participate in research is challenging. In recognition of this, we used a specialised fieldwork organisation and ethnically matched bilingual field staff. Illustrative of the challenges are our Chinese group where we extended the fieldwork by 3 months, approached potential participants and achieved a sample of 156, a response rate of 23%, but did not achieve our target of 200. Our study demonstrates the challenges of achieving our aspiration to include minority groups within mainstream gerontological research which need to be acknowledged by research funders and policy makers.

Our paper addressed three elements of a proposed gerontological research agenda focused upon the experiences of ageing and later life in minority communities: (a) determining the utility of standardised measures, in this case two established approaches to measuring loneliness, for use with older and mid-life adults from ethnic minority groups; (b) undertaking comparative research on loneliness prevalence across different ethnic groups in recognition that all minority communities are not homogeneous; and (c) investigating the degree to which ethnicity was an independent predictor of health and well-being outcomes using the example of loneliness. We review our success in meeting these three objectives in turn.

First in terms of the utility of our loneliness measures, we consider that the two measures used, the single-item question and six-item DJG scale, are appropriate for use with our six ethnic groups. Both measures had high levels of acceptability in a face-to-face interview with an ethnically matched interviewer as there were no missing data or interviews declined because of these questions. This does not, of course, establish acceptability for use by other data collection modes such as online or self-completion. We replicated the approaches of De Jong Gierveld and Van Tilburg (2010) and Uysal-Bozkir et al. (2017) in evaluating measure reliability and validity. This enables us to compare our results with those from other populations to see if there are emergent consistencies. Reliability, as measured by Cronbach’s alpha, for the total DJG measure and sub-scores showed moderate to high values (0.68–0.89) across all groups and were comparable to those reported for the eleven-item (0.78–0.90) (Uysal-Bozkir et al. 2017) and six-item versions (0.64) of the DJG scale (De Jong et al. (2015). Strong associations between the two dimensions of the DJG scale, the single-item loneliness question with the overall scale and sub-scales for all groups were observed with one exception which was social loneliness in the Black African group. A two-factor structure, differentiating the social and emotional domains of the overall scale, was confirmed for all groups. The CFA suggested a good fit for three groups, Black African, Indian and Pakistani, where all statistical criteria were meet, with two criteria meet for the remaining groups. This is, overall, a better statistical evaluation than that reported by Uysal-Bozkir et al. (2017) where only 1 of the 4 minority groups meet all 3 criteria. All estimated loadings for the individual scale items for the two-factor solution exceed 0.5 for all groups. However, it is noticeable that the loadings are lower across all groups on the emotional loneliness items which are al negatively orientated. In terms of structural validity, the total score and sub-scales for each group demonstrated significant correlations with the proposed congruent variable, well-being, and are in line with the work of Uysal-Bozkir et al. (2017). In contrast, weak mostly non-significant associations were observed with the non-congruent variables: number of languages spoken and responsibility for care of older adults.

We found only one study that evaluated the utility of the DJG scale in one of six ethnic groups and that was for Chinese participants. Leung et al. (2008) undertook a measure evaluation study of the six-item DJG scale using 103 community dwelling older adults. They report a correlation between the two DJG sub-scales of 0.37 compared to 0.48 in our study. The correlation between their overall loneliness scale, which asked if respondents were lonely with a yes/no response to which 20% replied yes, was 0.71 (0.61 in our study.). Overall, these findings broadly support the use of the DJG and a single-item loneliness question in our six specific ethnic groups.

Second, we sought to establish the prevalence of loneliness amongst our six ethnic groups. Drawing direct comparisons of our loneliness prevalence estimates with other studies of ethnic minority elders is problematic and is illustrative of the more general challenges in undertaking comparative gerontological work. Illustrate of the factors that need to be taken into account when making comparisons across studies include variations in the age structure of populations, variations between ethnic, linguistic and migrant statuses of populations studied, the use on country-based comparisons as proxies for ethnicity/culture, differences in loneliness measures used and variations in exposure to loneliness risk factors. We cannot draw direct comparisons of our substantive findings with Uysal-Bozkir et al. (2017) as they used the eleven-item DJG scale or with De Jong Gierveld and Tilburg (2010) as they included adults aged 18 + and is a comparison between different countries rather than between ethnic groups. Studies of the experiences of loneliness among minority groups are largely country specific, reflecting the migration patterns of specific groups. For example, research in The Netherlands and Germany has been based around the experiences of Turkish, Moroccan and Algerian migrants (see Klokgieters et al. 2019;2017; Visser and El Fakiriri 2016). In our study, we observed a consistent pattern across our different measures with Indian participants reporting the lowest levels of loneliness and Chinese the highest. This replicates the pattern previously reported by Victor et al. (2012) and mirrors the work from The Netherlands in demonstrating variations in loneliness prevalence across ethnic groups. Using responses to the single-item question, the proportion reporting that they were often or always lonely for those aged 65 + are much lower than in the previous study by Victor et al. (2012) (Black African (50% vs. 11%); Pakistani (50% vs. 16%); Bangladeshi (40% vs. 15%) and Chinese (40% vs. 25%) and Black Caribbean (24% vs. 16%) groups but comparable for the Indian group (8% v 7%). These discrepancies may reflect the pilot nature of the initial study and more robust sampling used in the current study, but may confirm that there are differences across ethnic groups in the reporting of loneliness. However, the consistently low level of loneliness reported by our Indian participants merits further investigation to (a) confirm that this is not a measurement artefact and (b) examine why loneliness is, at face value, less problematic for this population.

A comparative lens that just focuses upon ethnic groups within specific countries is, we argue, insufficient. We need to adopt comparative approaches including both other ethnic groups, but also native-born peers and peers in their country of origin using comparable measures of loneliness. Few studies have compared loneliness between migrants/minority elders and both native-born peers and those in their country of origin. Victor (2015) compared loneliness for Indian older adults, aged 65 + , living in Britain with their native-born peers and peers in India. This study showed that using a single-item rating scale, levels of loneliness, as defined by the percentage reporting that there were always/often lonely, were virtually identical across all three groups at eight to ten percent.

We can also make comparisons of loneliness prevalence for our Bangladeshi and Chinese population but not for the Pakistani, Black African or Black Caribbean groups. Rahman et al. (2020) report a prevalence of loneliness, using a score of 2 + on the six-item DJG scale for 400 aged 65 + in Meherpur district of Bangladesh of 54.5% which is comparable to the 62% in our study. There are studies of loneliness using the six-item DJG scale for Chinese elders in Hong Kong (Leung et al. (2008) and Chinese migrants to Australia (Lin et al. 2016). Leung et al. (2008) reported lower mean loneliness scores compared with our study (total score: 1.5 vs. 2.8; emotional loneliness: 0.6 vs. 1.4; social loneliness: 0.9 vs. 1.3). Lin et al. (8%) from Australia reported that 49% of their 59 Chinese migrant participants scored two or more on the six-item DJG scale compared with 61% in our study. These two comparative studies are suggestive of higher rates of loneliness in our sample of Chinese elders, but we must cautious in drawing robust inferences given the small sample sizes.

Three explanations may underpin the observed high rates of loneliness among five of our six ethnic minority groups included in this survey: measurement artefact; disproportionate exposure to loneliness vulnerability factors and sociocultural factors. Consistent with other studies, there does not seem to be a support for a solely measure artefact explanation. Fokkema and Naderi (2013) and Van Tilburg and Fokkema (2020) suggest that disproportionate exposure loneliness vulnerability factors may explain high levels of loneliness in minority communities. We explored this explanation using six established vulnerability factors selected to make direct comparison with the work of De Jong Gierveld and Van Tilburg (2010). In our initial modelling, three loneliness vulnerability factors were not associated with loneliness in our study—number of children, gender and health rating. This contrasts with the work although their analysis was a country-based study rather than ethnic group-based study. Three factors were significantly associated with loneliness: age, not being married and financial strain, with the latter two factors being ascribed greater statistical power than age. We tested the sociocultural explanation by running a second model that focused upon investigating the independent effect of ethnic group membership as a loneliness predictor. When ethnicity was added to the model, the independent predictors previously identified remained significant with only minor changes to the odds ratios and the addition of ethnicity did not enhance the power of the model. Membership of an ethnic group was protective against loneliness, but the relationship was only statistically significant for the African Caribbean group and the confidence interval is wide. This suggests that it is not ethnicity per se that is important in generating differences in loneliness across ethnic groups but other factors including exposure to loneliness vulnerabilities and other potential factors such length of residence in the UK, social networks, education and sense of belonging (see Klok et al. 2017) which were not included in our study.

## Conclusions

The population of Europe is both ageing and becoming more diverse, especially in terms of ethnicity. Gerontology needs to reflect this increasing diversity within our research agenda to ensure our work remains relevant and fully captures the experience of ageing within a European context. We suggest that there are five distinct elements to this research agenda: (a) the articulating within-group variations in terms of both temporal (cohort) and individual factors; (b) adopting an explicitly comparative perspective across ethnic groups and the experiences of specific populations across groups (e.g. women); (c) our comparative studies should be international in scope comparing, for example, Chinese populations who migrated to a range of different countries (e.g. the UK, Canada or Australia) and drawing comparisons with both those populations in the country of origin that did not migrate and peers in the host country; (d) our focus needs to extend beyond a pathological problem based focus; and (e) the development of methods and measures that are appropriate for use with diverse groups, which provide confidence that observed differences between/across groups are not an artefact or result of measurement error.

Using a sample of 1206 adults aged 40 + from six different ethnic groups, we have shown that the six-item DJG scale demonstrates satisfactory acceptability, reliability and validity and conclude that that differences in scores observed between groups do not simply reflect measurement issues. Levels of loneliness generated from the single-item loneliness question vary markedly from the DJG scale although the ordering of the groupings in terms of loneliness prevalence is broadly consistent. Further research is required to unpick the inter-relationships between these two measures and to consider the sub-domains of the DJG scale—emotional and social loneliness—in more detail. The negative orientation of the 3 emotional loneliness items may be problematic. There is also an important area of work in determining which type of loneliness measure is appropriate in different settings such as evaluating loneliness interventions, establishing the prevalence of loneliness and understanding and explaining the experiences of loneliness in different populations. In the UK, the development of a ‘common currency’ of loneliness measures is important, given the choice of a single-item loneliness question and the three-item UCLA scale as the national loneliness measures (ONS 2018).

The finding of consistently low (relative) loneliness rates across all measures for our Indian group is intriguing and merits further investigation both quantitatively and qualitatively as does the high levels of loneliness among Chinese participants. Our modelling, alongside that of Fokkema and Naderi (2013) and Klok et al. (2017), suggests that it may not be ethnicity per se that is responsible for elevated levels of loneliness among minority groups but rather exposure to loneliness vulnerability factors. We will explore these issues in more depth in our future work, given that we have established confidence in the utility of our measures.

## Electronic supplementary material

Below is the link to the electronic supplementary material.

Supplementary material 1 (DOCX 18 kb)

## Data Availability

The data underpinning this paper will be published online at the Brunel University Figshare data repository 10.177633/rd.brunel.11952318.
