# Supplementary material for: Loneliness in mid-life and older adults from ethnic minority communities in England and Wales: measure validation and prevalence estimates
Source: Eur J Ageing. 2020 Apr 7;18(1):5–16. doi: 10.1007/s10433-020-00564-9 (PMC7925782; doi:10.1007/s10433-020-00564-9)
Supplement: Supplementary file 1 — Supplementary material 1 (DOCX 18 kb) [file 10433_2020_564_MOESM1_ESM.docx]

Supplementary tables

Table S1

Self-rated loneliness by ethnicity (%)

| Scale | Black Caribbean | Black African | Indian | Pakistani | Bangladeshi | Chinese |
| --- | --- | --- | --- | --- | --- | --- |
| N | 224 | 215 | 201 | 211 | 199 | 156 |
| Never | 25 | 22 | 57 | 33 | 28 | 21 |
| Rarely | 33 | 39 | 24 | 28 | 32 | 30 |
| Sometimes | 30 | 32 | 14 | 28 | 29 | 35 |
| Often | 11 | 6 | 5 | 10 | 11 | 13 |
| Always | 1 | 1 | 0 | 1 | 0 | 1 |

Table S2 Self-rated loneliness by ethnicity and age (%)

| Scale | Black Caribbean | | Black African | | Indian | | Pakistani | | Bangladeshi | | Chinese | |
| --- | --- | --- | --- | --- | --- | --- | --- | --- | --- | --- | --- | --- |
| Age | 40-64 | 65+ | 40-64 | 65+ | 40-64 | 65+ | 40-64 | 65+ | 40-64 | 65+ | 40-64 | 65+ |
| N | 104 | 120 | 112 | 103 | 98 | 103 | 103 | 108 | 99 | 100 | 90 | 66 |
| Never | 32 | 20 | 30 | 14 | 55 | 59 | 43 | 21 | 34 | 22 | 29 | 9 |
| Rarely | 31 | 34 | 36 | 42 | 25 | 23 | 25 | 31 | 28 | 35 | 30 | 30 |
| Sometimes | 31 | 30 | 30 | 34 | 17 | 11 | 25 | 30 | 30 | 28 | 34 | 36 |
| Often | 7 | 15 | 3 | 10 | 3 | 7 | 5 | 14 | 7 | 14 | 6 | 21 |
| Always | 0 | 1 | 1 | 1 | 0 | 0 | 2 | 2 | 0 | 1 | 0 | 3 |

Table S3 Mean DJG score and sub-scale scores by ethnicity and age

| Scale | Black Caribbean | | Black African | | Indian | | Pakistani | | Bangladeshi | | Chinese | |
| --- | --- | --- | --- | --- | --- | --- | --- | --- | --- | --- | --- | --- |
| Age | 40-64 | 65+ | 40-64 | 65+ | 40-64 | 65+ | 40-64 | 65+ | 40-64 | 65+ | 40-64 | 65+ |
| N | 104 | 120 | 112 | 103 | 98 | 103 | 103 | 108 | 99 | 100 | 90 | 66 |
| Mean total loneliness score | 2.4 | 2.7 | 2.5 | 2.9 | 1.8 | 2.0 | 1.8 | 3.0 | 2.4 | 2.8 | 2.4 | 3.2 |
| Mean emotional loneliness score | 1.0 | 1.3 | 0.8 | 1.3 | 0.7 | 0.7 | 0.7 | 1.3 | 1.0 | 1.1 | 1.1 | 1.6 |
| Mean social loneliness score | 1.3 | 1.4 | 1.6 | 1.6 | 1.0 | 1.2 | 1.0 | 1.7 | 1.3 | 1.6 | 1.3 | 1.6 |
| % with DJG score of 2+ | 56.7 | 55.0 | 66.1 | 74.0 | 54.8 | 51.5 | 47.0 | 69.0 | 57.3 | 67.0 | 54.3 | 69.7 |
| % with a DJG score of 5+ | 21.1 | 35.9 | 15.1 | 24.3 | 10.2 | 17.5 | 13.4 | 35.2 | 21.3 | 27.0 | 27.8 | 45.5 |

Table S4: Logistic Regression Equation, predicting Loneliness group (without Ethnicity). Loneliness group based on self rated loneliness “Always” or “Often”. Significant predictor results in bold. (N= 1206)

Predictor B S.E. Wald *df*  *p* Odds Ratio 95% C.I

Total children .39 .21 3.36 1 .067 1.48 [.97, 2.24]

**Age** -.04 .01 10.47 1 .001 .97 [.95, .99]

Gender -.17 .21 .66 1 .42 .84 [.56, 1.27]

**Married**  1.03 .21 23.20 1 .000 2.80 [1.84, 4.25]

Health rated good .58 .26 5.21 1 .022 1.80 [1.08, 2.95]

**Financial strain**  1.20 .21 32.56 1 .000 3.33 [2.21, 5.05]

____________________________________________________________________

χ^2^ (6) = 110.254**, Nagelkerke *R*^2^ = .19

Coding Key: Loneliness Group ( 1 = not lonely; 0 = lonely); Total children (1 = 3 or more children; 0 = 2 or fewer); Gender (1= male; 2 = female); Age (in years); Married (1 = yes; 0 = no); Health rating ( 1 = good; 0 = not good); Financial strain (1= not strained; 0 = strained)

Reference group=Chinese

Table S5: Logistic Regression Equation, predicting Loneliness group (with Ethnicity as a dummy variable, Reference group Chinese). Loneliness group based on self rated loneliness “Always” or “Often”.Significant predictor results in bold.

Predictor B S.E. Wald *df* *p* Odds Ratio 95% C.I

Total children .32 .22 2.01 1 .156 1.37 [.89, 2.13]

**Age** -.03 .01 8.78 1 .003 .97 [.95, .99]

Gender -.13 .21 .38 1 .540 .88 [.58, 1.33]

**Married**  1.10 .22 25.31 1 .000 3.02 [1.96, 4.65]

Health rating .63 .26 5.89 1 .015 1.88 [1.13, 3.14]

**Financial strain**  1.18 .22 28.37 1 .000 3.28 [2.12, 5.07]

Ethnicity 9.55 5 .089

Black African .46 .35 1.78 1 .182 1.58 [.81, 3.11]

**Caribbean African** 1.03 .39 6.96 1 .008 2.80 [1.30, 6.02]

Indian .79 .43 3.31 1 .070 2.19 [.94, 5.10]

Pakistani .17 .36 .24 1 .625 1.19 [.59, 2.39]

Bangladeshi .49 .38 1.71 1 .190 1.64 [.78, 3.42]

_____________________________________________________________________

Coding Key: Loneliness Group ( 1 = not lonely; 0 = lonely); Total children (1 = 3 or more children; 0 = 2 or fewer); Gender (1= male; 2 = female); Age (in years); Married (1 = yes; 0 = no); Health rating ( 1 = good; 0 = not good); Financial strain (1= not strained; 0 = strained)

Reference group=Chinese

χ^2^ (11) =120.195***, Nagelkerke *R*^2^ = .202.

Improvement in fit, *with* Ethnicity *v*. *without* Ethnicity, χ2 (5) = 9.94, ns.
